# Requirement for PLK1 kinase activity in the maintenance of a robust spindle assembly checkpoint

**DOI:** 10.1242/bio.014969

**Published:** 2015-12-18

**Authors:** Aisling O'Connor, Stefano Maffini, Michael D. Rainey, Agnieszka Kaczmarczyk, David Gaboriau, Andrea Musacchio, Corrado Santocanale

**Affiliations:** 1 Centre for Chromosome Biology, School of Natural Sciences, National University of Ireland Galway, Galway, Ireland; 2 Max-Planck Institute of Molecular Physiology, Department of Mechanistic Cell Biology, Dortmund 44227, Germany; 3 Centre for Medical Biotechnology, Faculty of Biology, University Duisburg-Essen, Universitätsstrasse, Essen 45141, Germany

**Keywords:** Cell-Cycle, PLK1, Aurora B, MPS1, Haspin, Spindle assembly checkpoint, Kinase inhibitors

## Abstract

During mitotic arrest induced by microtubule targeting drugs, the weakening of the spindle assembly checkpoint (SAC) allows cells to progress through the cell cycle without chromosome segregation occurring. PLK1 kinase plays a major role in mitosis and emerging evidence indicates that PLK1 is also involved in establishing the checkpoint and maintaining SAC signalling. However, mechanistically, the role of PLK1 in the SAC is not fully understood, with several recent reports indicating that it can cooperate with either one of the major checkpoint kinases, Aurora B or MPS1. In this study, we assess the role of PLK1 in SAC maintenance. We find that in nocodazole-arrested U2OS cells, PLK1 activity is continuously required for maintaining Aurora B protein localisation and activity at kinetochores. Consistent with published data we find that upon PLK1 inhibition, phosphoThr3-H3, a marker of Haspin activity, is reduced. Intriguingly, Aurora B inhibition causes PLK1 to relocalise from kinetochores into fewer and much larger foci, possibly due to incomplete recruitment of outer kinetochore proteins. Importantly, PLK1 inhibition, together with partial inhibition of Aurora B, allows efficient SAC override to occur. This phenotype is more pronounced than the phenotype observed by combining the same PLK1 inhibitors with partial MPS1 inhibition. We also find that PLK1 inhibition does not obviously cooperate with Haspin inhibition to promote SAC override. These results indicate that PLK1 is directly involved in maintaining efficient SAC signalling, possibly by cooperating in a positive feedback loop with Aurora B, and that partially redundant mechanisms exist which reinforce the SAC.

## Abstract (precis)

**Summary:** Using small molecule kinase inhibitors we find that PLK1 and Aurora B cooperate to avoid mitotic slippage upon microtubule depolymerization and to promote their reciprocal recruitment at kinetochores.

## INTRODUCTION

Anticancer drugs that interfere with microtubule dynamics cause a prolonged cell cycle arrest in mitosis due to sustained activation of the spindle assembly checkpoint (SAC). Eventually cells either die during the mitotic arrest or override the drug-induced cell cycle arrest and exit mitosis without undergoing chromosome segregation, a phenomenon defined as mitotic slippage (often also defined as adaptation) (Gascoigne and Taylor, 2008; Topham and Taylor, 2013). The duration of the induced cell cycle arrest is dependent on the robustness of the SAC, a complex feedback control mechanism that prevents metaphase to anaphase progression until all kinetochores are properly attached to the microtubules of the mitotic spindle. When bipolar kinetochore microtubule attachment (k-MT) occurs, inhibition of the CDC20 co-activator of the ubiquitin E3 ligase anaphase promoting complex/cyclosome (APC/C) is relieved, leading to polyubiquitination and degradation of Cyclin B and securin which promotes anaphase and mitotic exit (Musacchio and Salmon, 2007). Thus, the SAC normally ensures faithful chromosome segregation and genome stability and it is an important determinant of cell fate after chemotherapy based on drugs that affect microtubules (Janssen et al., 2009).

Signalling pathways usually consist of three phases: establishment, maintenance and silencing. The establishment of the SAC has been widely studied; many factors participate in and regulate SAC establishment at the kinetochore, including Aurora B, MPS1 and Haspin protein kinases. In the establishment phase, Haspin promotes Histone H3 phosphorylation at Thr 3 (Dai et al., 2005). This in turn allows recruitment of the chromosomal passenger complex (CPC) and Aurora B activation (Wang et al., 2010). The CPC consists of Aurora B kinase and its regulatory proteins, Survivin, INCENP and Borealin and it is required for the activation and the maintenance of the SAC (Carmena et al., 2012b). Aurora B together with the kinetochore protein HEC1 allows the recruitment and activation of MPS1, which is important for the kinetochore recruitment of other proteins, including PLK1, MAD1 and MAD2, that are required for complete activation of the checkpoint (Musacchio and Salmon, 2007; Saurin et al., 2011). MPS1 has also been reported to further activate Aurora B through phosphorylation of Borealin (Jelluma et al., 2008). SAC components MAD2, BUB3 and BUBR1 form a sub-complex called the mitotic checkpoint complex (MCC), which is a SAC effector. The MCC prevents CDC20 activation of the APC/C and thus prevents anaphase onset until all k-MT attachments are bipolar and stable (Sudakin et al., 2001).

The same kinases which are required for establishment are also clearly involved in the maintenance of the checkpoint. Direct inhibition of SAC kinases, such as MPS1 (Santaguida et al., 2010), Aurora B (Hauf et al., 2003; Santaguida et al., 2011) or Haspin (De Antoni et al., 2012) can abruptly override SAC induced cell-cycle arrest. In particular, Aurora B, MPS1 and Haspin small molecule inhibitors, Hesperadin, Reversine and 5-iodutubercidin, respectively, can cause mitotic slippage of nocodazole arrested cells either as single agents or synergistically when used in combinations at suboptimal concentrations, thus defining distinct control nodes in this pathway (Santaguida et al., 2011; Saurin et al., 2011; Wang et al., 2012).

Polo-like kinase 1 (PLK1) plays many roles leading to cell division including promoting entry into, progression through and exit from mitosis (Lindon and Pines, 2004; van Vugt and Medema, 2005). It regulates bipolar spindle formation, centrosome function and k-MT attachment in human cells (Lenart et al., 2007; Peters et al., 2006).

Recent work is also beginning to define a role for PLK1 in the SAC. The function of the PLK1 *Drosophila* orthologue Polo at the kinetochore was shown to be regulated by Aurora B dependent phosphorylation of its activation loop (Carmena et al., 2012a), where Polo functions upstream of MPS1, allowing MPS1 recruitment to the kinetochore (Conde et al., 2013). In human cells instead it was reported that PLK1 phosphorylates Haspin thus stimulating Histone H3 phosphorylation at Thr 3 and contributing to Aurora B kinetochore recruitment (Zhou et al., 2014). Furthermore, Aurora B activity at the centromere is regulated by PLK1 through a survivin priming phosphorylation event (Chu et al., 2011).

Inhibition of PLK1, unlike the inhibition of Haspin, Aurora B and MPS1, is not sufficient to override the SAC induced cell cycle arrest, indicating that PLK1 is not strictly essential for the checkpoint. The biological relevance of PLK1 kinase in maintaining and activating the SAC was only uncovered by inhibiting PLK1 while also partially inhibiting Aurora B (Li et al., 2015). A recent report indicated that the major targets of PLK1 during SAC maintenance are a set of proteins that are also MPS1 targets, including KNL-1 and MELT (von Schubert et al., 2015). PLK1 cooperates with MPS1 in the establishment and maintenance of the SAC in RPE-1 cells and the combined inhibition of these kinases causes a SAC override. However, the role of PLK1 in SAC maintenance is controversial. A recent publication has shown that cells arrested in mitosis with PLK1 inhibitors have low levels of Aurora B at kinetochores (Raab et al., 2015). Another recent publication instead showed that PLK1 inhibitors do not affect Aurora B localisation (von Schubert et al., 2015).

In this work, using two chemically unrelated PLK1 small molecule inhibitors, we evaluate the role of PLK1 in the maintenance of Aurora B at kinetochores in U2OS cells, a widely used cellular model; we also assess the effects of the PLK1 inhibitors together with partial inhibition of the three major checkpoint kinases Aurora B, MPS1 and Haspin in maintaining the strength of the nocodazole induced mitotic arrest.

## RESULTS

### Maintenance of Aurora B at kinetochores and CENP-A phosphorylation in nocodazole treated cells requires PLK1 activity

**Fig. 1.**
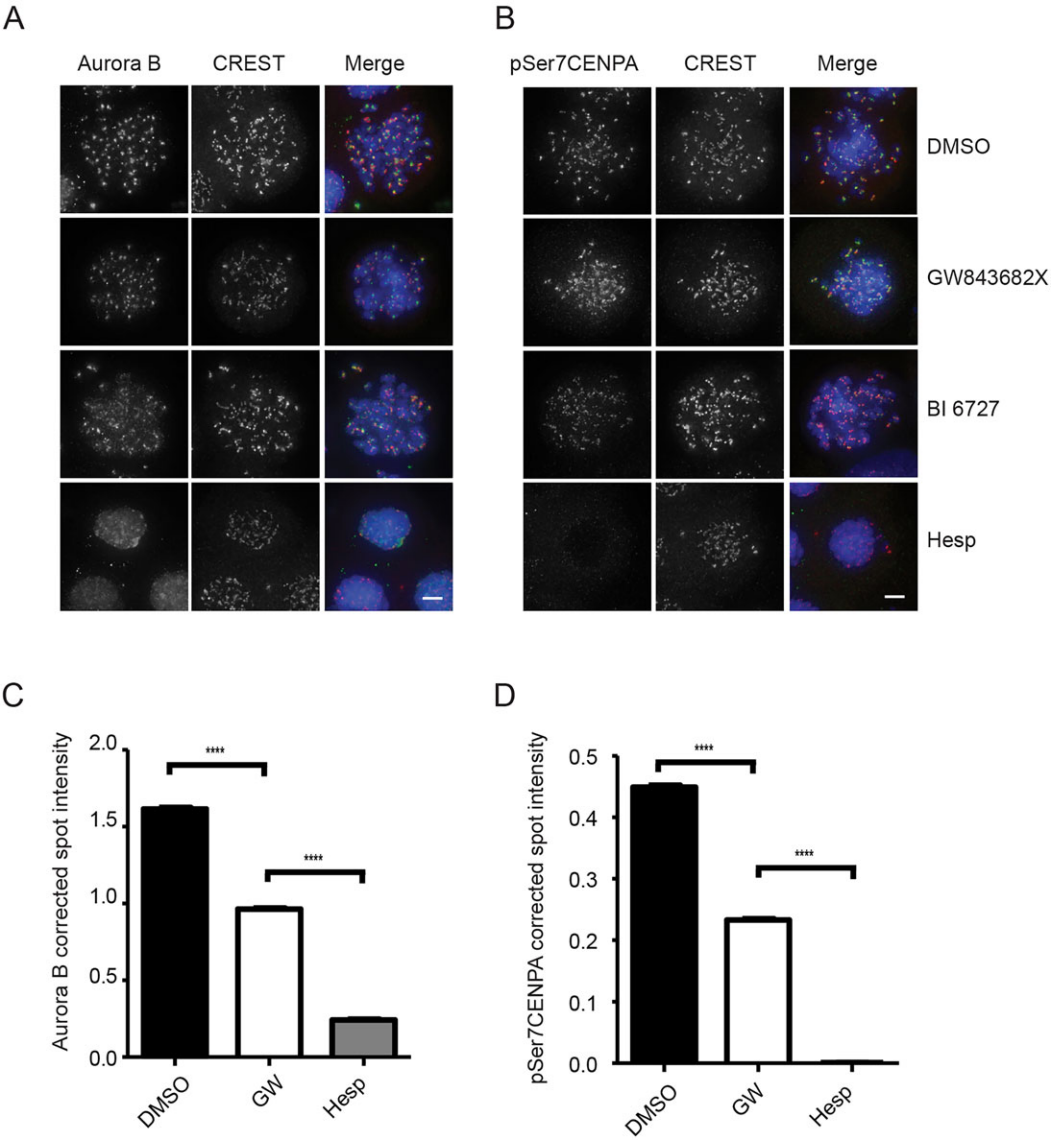
**PLK1 inhibitors decrease Aurora B and pSer7CENP-A levels at the kinetochores.** (A,B) U2OS cells were arrested in nocodazole for 12 h, collected by mitotic shake-off and re-plated in the presence of nocodazole and MG132 with either 1 μM GW843682X, 100 nM BI 6727, 500 nM Hesperadin or DMSO for 3 h. Cells were stained with (A) anti-Aurora B (green), CREST (red) and DAPI (blue), (B) anti-pSer7CENP-A (green), CREST (red) and DAPI (blue) for immunofluorescence. Scale bar: 5 μm. (C,D) Quantification of Aurora B and pSer7CENP-A spot intensity in control, GW843682X- and Hesperadin-treated samples were performed using a High Content Operetta system. Aurora B and pSer7CENP-A corrected spot intensity were normalised to CREST. At least 680 mitotic cells per treatment were included in the counts, and the number of CREST spots was at least 13,900 per treatment per repeat. Error bar represent s.e.m. and ****P<0.0001. One-tailed *t*-test was used.

Due to the controversy about the function of PLK1 in SAC maintenance, we set out to independently determine if the maintenance of Aurora B at kinetochores requires continuous PLK1 activity in U2OS cells upon complete disruption of microtubules by high doses of nocodazole. In our experiments, cells were treated with 3.3 μM nocodazole for 12 h, followed by treatment with either one of two chemically unrelated PLK1 inhibitors, GW843682X (Lansing et al., 2007) or BI 6727 (also known as Volasertib) (Rudolph et al., 2009) in the presence of proteasome inhibition to retain cells in mitosis. After 3 h of inhibition, cells were fixed and stained with anti-Aurora B antibodies and co-stained with CREST in order to mark the position of kinetochores. In control cells Aurora B is clearly detectable at kinetochores, while the addition of either GW843682X or BI 6727 caused a partial decrease in Aurora B intensity at the kinetochore with an overall more diffuse staining pattern (Fig. 1A). The decrease in Aurora B intensity and the diffuse localisation of Aurora B in the presence of PLK1 inhibitors was obvious with BI 6727 treatment but less marked with GW843682X. As a positive control Aurora B localisation at kinetochores was almost completely lost when cells were challenged with high doses of the Aurora B inhibitor Hesperadin (Hauf et al., 2003) (Fig. 1A).

To investigate the effect of PLK1 inhibition on the activity of Aurora B at kinetochores, cells were immunostained with antibodies against phosphorylated CENP-A at Ser7 (pSer7CENP-A), an established intracellular marker of Aurora B activity (Zeitlin et al., 2001). As shown in Fig. 1B, pSer7CENP-A is decreased in the presence of GW843682X, more evidently with BI 6727 and almost completely abolished upon treatment with Hesperadin.

Since the effects of GW843682X on both Aurora B and pSer7CENP-A staining at the kinetochore were less severe than the effects caused by BI 6727, we performed quantitative image analysis on the cells treated with GW843682X using the Perkin-Elmer Operetta high-content system. Briefly, the intensity of Aurora B staining at each kinetochore was normalised to the intensity of the corresponding CREST spot (see Materials and Methods). In this manner, by analysing over 6000 foci in over 600 cells, we confirmed that Aurora B intensity at the kinetochore was decreased upon GW843682X treatment (Fig. 1C). Similarly, the analysis of pSer7CENP-A corrected spot intensity normalised to CREST confirmed that there is a significant decrease in Aurora B activity at kinetochores (Fig. 1D).

Thus PLK1 inhibitors affect the maintenance of Aurora B localisation and activity at kinetochores in U2OS cells.

### PLK1 and Aurora B inhibition reduce pT3H3 levels

**Fig. 2.**
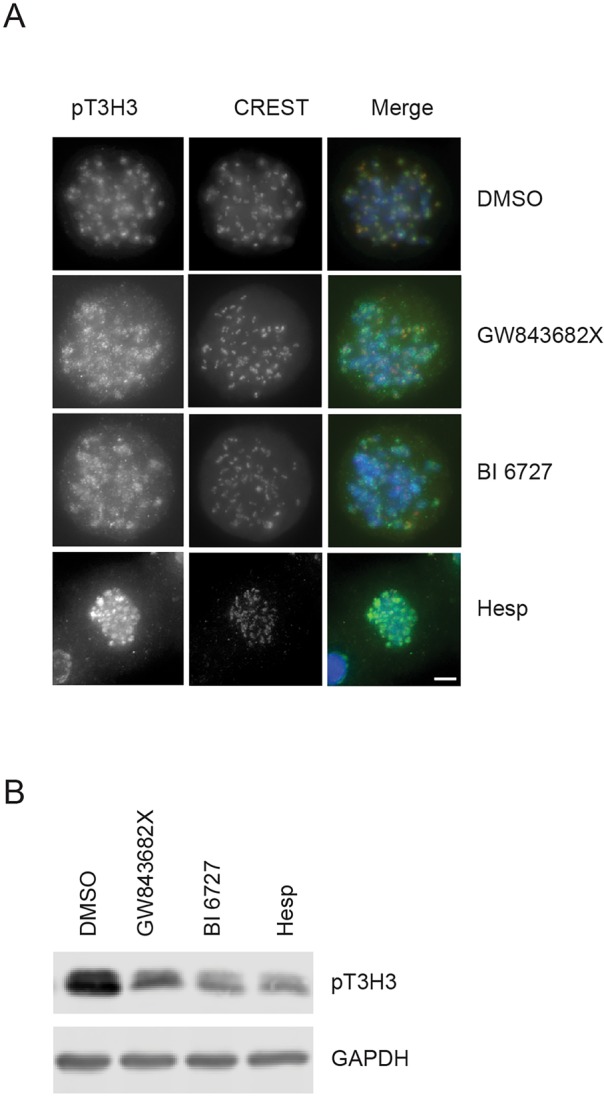
**PLK1 inhibitors and an Aurora B inhibitor reduce pT3H3 levels.** (A) Nocodazole-arrested cells were treated with either 1 μM GW843682X, 100 nM BI 6727, 500 nM Hesperadin or DMSO for 3 h in the presence of MG132 and nocodazole. Cells were stained with anti-pT3H3 (green), CREST (red) antibodies and DAPI (blue). Scale bar: 5 μm. (B) Cells were treated with nocodazole as above followed by shake-off and re-plated in the presence of nocodazole and 1 μM GW843682X, 100 nM BI 6727 or 500 nM Hesperadin, or DMSO and MG132 for 3 h and proteins were analysed by western blot with the indicated antibodies.

A possible mechanism by which Aurora B may be partially delocalised from kinetochores upon PLK1 inhibition could be accounted for by lower Haspin activity and loss of phosphorylation of histone H3 on Thr 3 (pT3H3) which contributes to Aurora B recruitment (Dai et al., 2005; Wang et al., 2010; Ghenoiu et al., 2013; Zhou et al., 2014). We tested this hypothesis under our experimental conditions. Cells were arrested in mitosis with nocodazole and treated with either PLK1 inhibitors or the Aurora B inhibitor Hesperadin and stained with anti-pT3H3 antibodies and CREST. We indeed observed that pT3H3 co-localisation with CREST is partially impaired when cells are treated with the PLK1 inhibitors and the signal of pT3H3 is less intense and more dispersed compared to the control. In cells treated with Hesperadin as a positive control, the loss of pT3H3 from kinetochores was more severe (Fig. 2A). To investigate if pT3H3 was simply delocalised or if the overall levels were reduced, western blot analysis was carried out. Fig. 2B shows that pT3H3 levels are reduced upon treatment with both PLK1 inhibitors GW843682X and BI 6727 compared to the control. Similarly, Hesperadin treatment reduces pT3H3 (Wang et al., 2011). Thus, PLK1 activity appears to be required for continuous maintenance of pT3H3 levels and its accumulation at kinetochores.

### Both PLK1 and Aurora B inhibitors cause PLK1 and outer kinetochore proteins to mis-localise

**Fig. 3.**
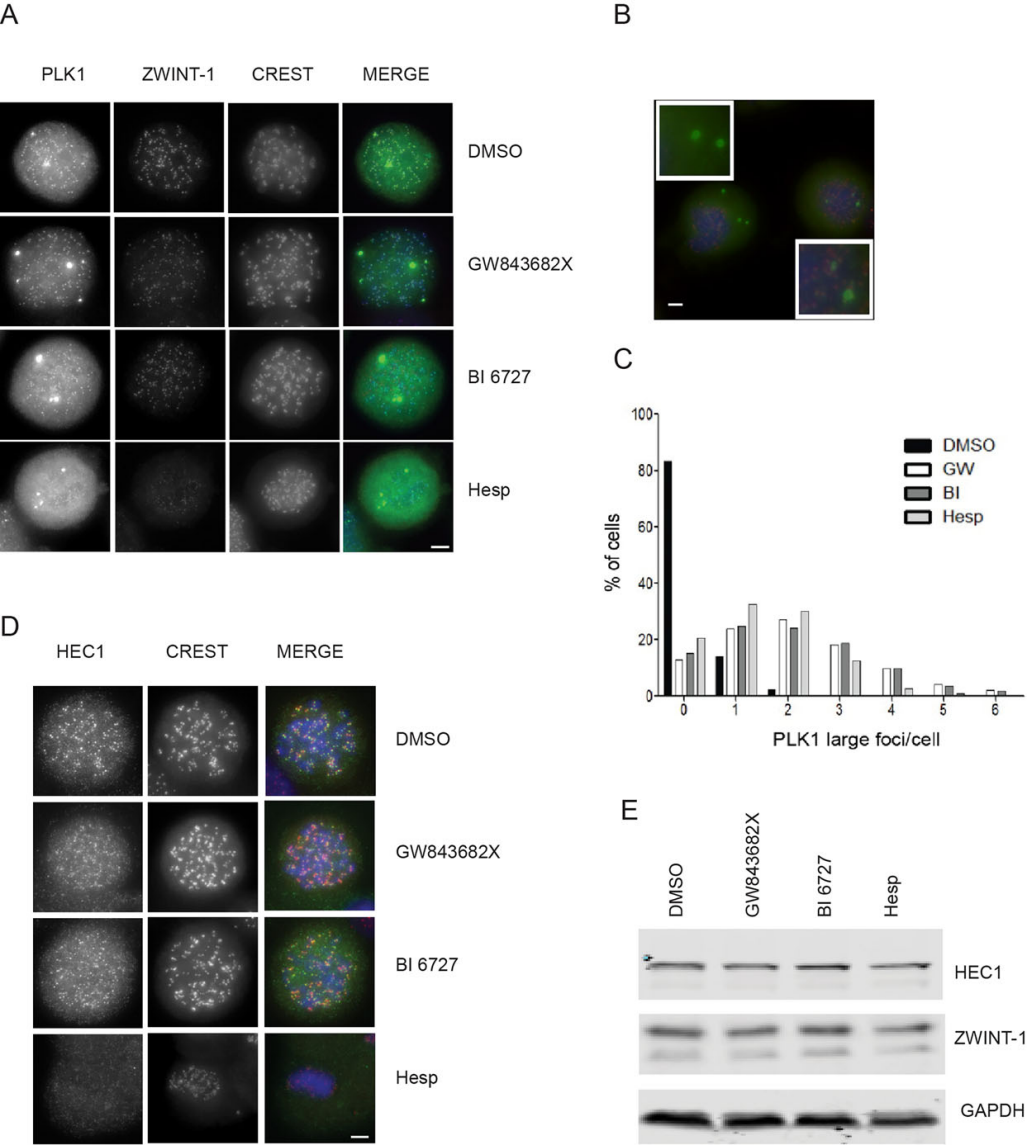
**PLK1 inhibitors and an Aurora B inhibitor affect PLK1 localisation and the localisation of HEC1 and ZWINT-1 at kinetochores.** Nocodazole-arrested cells were treated with either 1 μM GW843682X, 100 nM BI 6727, 500 nM Hesperadin or DMSO for 3 h in the presence of MG132 and nocodazole. Cells were stained with (A) anti-PLK1 (green), anti-ZWINT-1 (red) and CREST (blue) antibodies. Scale bar: 5 μm. (B) Representative image of Hesperadin-treated cells co-stained with anti-PLK1 (green) anti-CREST (red) antibodies and DAPI (blue). Scale bar: 5 μm. (C) Distribution of mitotic cells with large PLK1 foci after treatment with GW843682X, BI 6727, Hesperadin or DMSO as in A. At least 625 mitotic cells were counted for each treatment per repeat. (D) Cells were treated as in A and stained with anti-HEC1 (green), CREST (red) and DAPI (blue). Scale bar: 5 μm. (E) Cells were treated with nocodazole as above followed by shake-off and re-plated in the presence of nocodazole and 1 μM GW843682X, 100 nM BI 6727, 500 nM Hesperadin or DMSO for 3 h and in the presence of MG132. Proteins were analysed by western blot with the indicated antibodies.

PLK1 recruitment to kinetochores is required to regulate proper chromosome alignment and its removal is important for checkpoint silencing (Liu et al., 2012). To test whether PLK1 and Aurora B inhibitors affect PLK1 localisation during checkpoint maintenance, cells were arrested in mitosis with nocodazole and treated with Aurora B or with PLK1 inhibitors and stained with anti-PLK1 antibodies. In all cases we observed that normal kinetochore localisation of PLK1 is no longer detected but that the protein accumulates in fewer and larger foci (Fig. 3A-C). We also observed that these larger PLK1 foci are often in proximity to CREST but occasionally are also found outside of chromosomes indicating that these are unlikely to represent abnormal kinetochore recruitment of PLK1 (Fig. 3B).

It is known that Aurora B is required for the recruitment of outer kinetochore proteins (Emanuele et al., 2008). To understand if the effect of PLK1 or Aurora B inhibition on their localisation during maintenance also correlates with errors in the recruitment of outer kinetochore proteins, we arrested cells in mitosis and treated them with inhibitors as before. Cells were then stained with anti-PLK1 and anti-ZWINT1-1 antibodies. Indeed both GW843682X and BI 6727 decrease ZWINT-1 kinetochore levels and ZWINT-1 foci were not detected upon Hesperadin treatment (Fig. 3A). Similarly, Fig. 3D and Fig. S1 show that upon PLK1 inhibition with GW843682X and BI 6727, HEC1 is mislocalised. Western blot analysis indicates that overall HEC1 and ZWINT-1 proteins levels are not affected upon treatment with PLK1 or Aurora B inhibitors compared to the control (Fig. 3E). Our results are consistent with published data which demonstrates that outer kinetochore protein localisation is affected upon treatment with a combination of low doses of two different Aurora B inhibitors under different experimental conditions (Emanuele et al., 2008). We add to this that HEC1 and ZWINT-1 proteins are mis-localised in the presence of PLK1 inhibition under these experimental conditions.

**Fig. 4.**
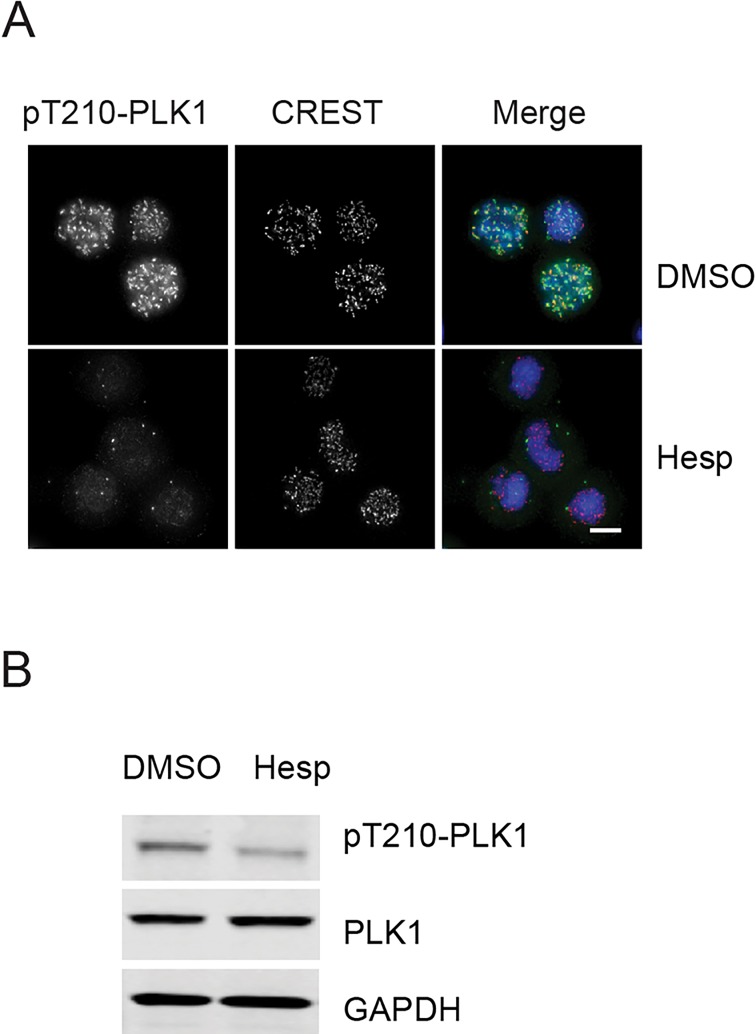
**Aurora B inhibitor reduces pT210-PLK1 levels.** (A) Cells were treated as in Fig. 3A and stained with pT210-PLK1 (green), CREST (red) and DAPI (blue). Scale bar 10 μm. (B) Cells were treated as in Fig. 3E and proteins were analysed by western blot with the indicated antibodies.

Another mechanism, which is important for establishment of the SAC involves phosphorylation of the Polo T-loop. In *Drosophila* cells Aurora B has been shown to phosphorylate Polo at T182 (corresponding to human PLK1 T210) and this is essential for Polo function at the kinetochore (Carmena et al., 2012a). To test whether Aurora B inhibitors affect PLK1 activity during checkpoint maintenance, cells were arrested in mitosis with nocodazole, treated with the Aurora B inhibitor Hesperadin and stained with phospho-specific antibodies recognising T210-PLK1 phosphorylation and CREST. pT210-PLK1 staining showed a similar altered pattern to PLK1 when treated with an Aurora B inhibitor (Fig. 4A). Western blot analysis also indicated that the overall levels of pT210-PLK1 phosphorylation were reduced upon Hesperadin treatment (Fig. 4B).

These results suggest that PLK1 and Aurora B may cooperate with each other in a positive feedback loop to regulate their localisation and activity during a prolonged cell cycle arrest in mitosis.

### PLK1 inhibitors weaken the spindle assembly checkpoint

Having established that PLK1 inhibitors affect recruitment and activity of both PLK1 and Aurora B at kinetochores, we asked if this would have consequences for SAC function. In particular, we tested whether PLK1 inhibition would result in override of the SAC under conditions in which SAC signalling is partially compromised.

**Fig. 5.**
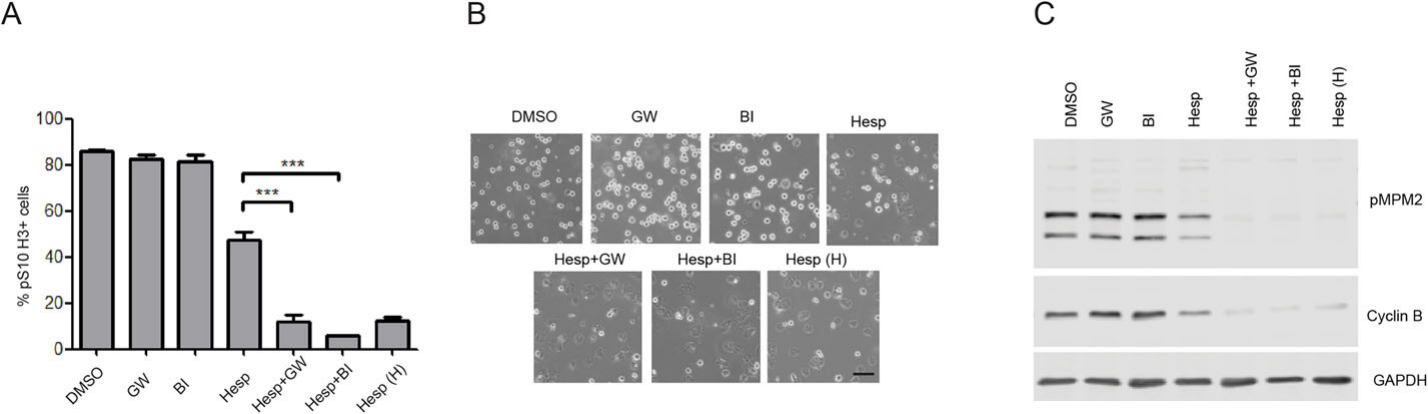
**PLK1 inhibitors and Aurora B inhibitor cooperate to weaken the checkpoint.** (A) Cells were treated with nocodazole for 12 h, collected by mitotic shake-off and re-plated in the presence of nocodazole minus/plus 1 μM GW843682X (GW), 100 nM BI 6727 (BI), 100 nM Hesperadin, both 1 μM GW843682X and 100 nM Hesperadin (Hesp+GW), both 100 nM BI 6727 and 100 nM Hesperadin (Hesp+BI), or 500 nM Hesperadin (Hesp H) as positive control. Cells were then collected, stained with anti-Histone H3 pSer10 antibody and analysed by FACS. 10,000 cells were counted for each treatment per repeat. Error bars are s.d. ***P<0.001, *N*=3. One-tailed *t*-test was used. (B) DIC images of A captured at the time of harvesting the cells. Scale bar: 50 μm. (C) Cells were treated as in A and western blot analysis was carried out using the indicated antibodies.

Cells were treated with nocodazole for 12 h, mitotic cells were collected by shake-off and re-plated in the continued presence of nocodazole. PLK1 inhibitors were then added either as single agents or together with suboptimal doses of the Aurora B inhibitor, Hesperadin. After 3 h cells were stained with antibodies against the mitotic marker pSer10 Histone H3 and scored using flow cytometry. Fig. 5A shows that more than 80% of the cells in the DMSO treated control sample were pSer10 Histone H3 positive and that PLK1 inhibitors GW843682X or BI 6727 did not change this outcome while high doses of Hesperadin (500 nM) used as a positive control resulted in the disappearance of pSer10 Histone H3 positive cells. A low dose of Hesperadin (100 nM) only caused reduction from approximately 85% to approximately 45% in the number of pSer10 Histone H3 positive cells. Since Aurora B can also phosphorylate Histone H3 on Ser10 we verified by FACS that the low levels of Hesperadin used do not reduce the intensity of Histone H3 pSer10 in the remaining fraction of Histone H3 pSer10 positive cells (Fig. S2). Importantly, the addition of PLK1 inhibitors in combination with a low dose of Hesperadin almost completely abolished Histone H3 pSer10 staining.

In samples treated with a combination of PLK1 inhibitors and a low dose of Hesperadin we saw that the reduction of pSer10 Histone H3 positive cells closely correlated with the number of cells that lost the typical round shaped morphology of mitotic cells and had reattached to the plates (Fig. 5B) suggesting that in these cells the mitotic cell cycle arrest was overridden. Western blot analysis of protein extracts revealed that both mitotic markers pMPM2 and Cyclin B levels were dramatically reduced in the presence of combined inhibition of PLK1 and Aurora B compared to the control or when PLK1 inhibitors or low levels of Hesperadin were used as single agents confirming that these cells have slipped out of mitosis. Altogether these results indicate that PLK1 and Aurora B inhibition cooperate to cause a SAC override driving the cells into a G1 like state.

**Fig. 6.**
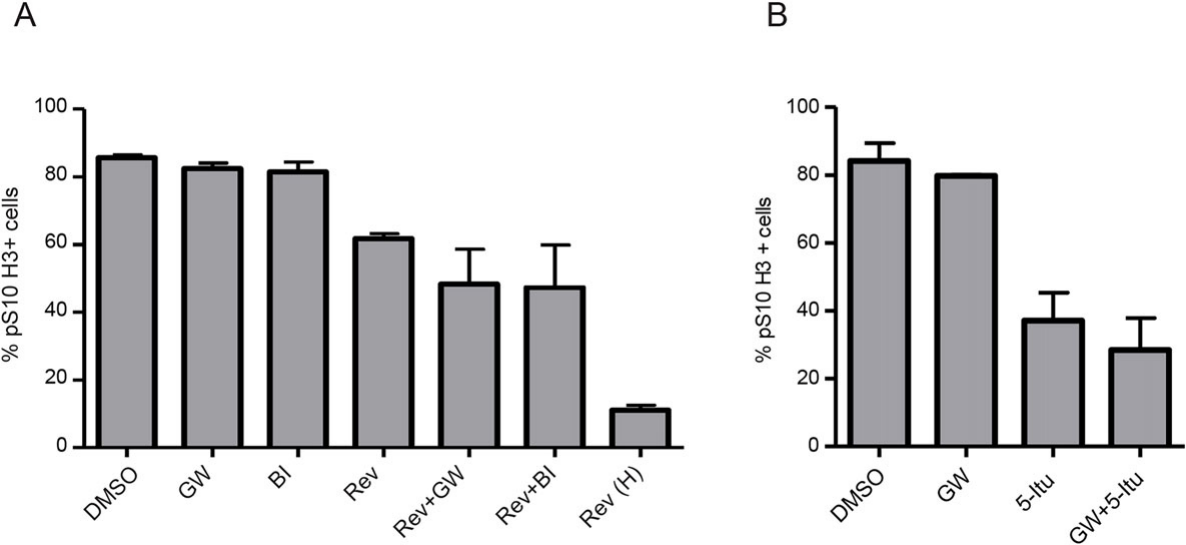
**Effect of PLK1 inhibitors in combination with inhibitors of MPS1 or Haspin.** Nocodazole-arrested cells were re-plated in the presence of nocodazole (A) with or without 1 μM GW843682X (GW), 100 nM BI 6727 (BI) or 100 nM Reversine (Rev), both GW843682X and 100 nM Reversine (Rev+GW), both BI 6727 and 100 nM Reversine (Rev+BI), or high dose (500 nM) Reversine [Rev (H)]. After 3 h cells were collected, stained and analysed by flow cytometry. 10,000 cells were counted for each treatment per repeat. Error bars are s.d., *N*=3. (B) Nocodazole-arrested cells were re-plated in the presence of nocodazole with or without 1 μM GW843682X (GW), 10 μm 5-Itu or both GW843682X and 5-Itu (GW+5-Itu). Cells were then stained and analysed as in A. Error bars are s.d., *N*=2.

With a similar strategy we assessed if PLK1 inhibitors can override the SAC when other checkpoint kinases are partial inhibited, namely MPS1 and Haspin. Unlike with Hesperadin, the combination of GW843682X or BI 6727 with low doses of the MPS1 inhibitor Reversine did not show a statistically significant increase in the fraction of pSer10 Histone H3 negative cells compared to Reversine alone (Fig. 6A). Similar to Reversine, we did not observe a statistically significant increase in the percentage of pSer10 Histone H3 negative cells when GW843682X was combined with low levels of the Haspin inhibitor 5-Itu compared to low levels of 5-Itu alone (Fig. 6B).

These results indicate that upon disruption of microtubules in U2OS cells PLK1 functions to cooperate with Aurora B to maintain a mitotic cell cycle arrest.

## DISCUSSION

In this work we have assessed the effects of PLK1 inhibitors on the localisation of key SAC proteins and their effects as single agents, or in combination, on the maintenance of the mitotic cell cycle arrest in nocodazole treated cells. We have independently confirmed that PLK1 contributes to the maintenance of the SAC and that PLK1 inhibition affects the localisation and activity of Aurora B at kinetochores. PLK1 activity is not only required to establish Aurora B localisation as previously shown (Raab et al., 2015) but it is also continuously required for its maintenance, at least in U2OS cells. This is different from what has been reported by another group that have shown that PLK1 activity is not required for recruitment of Aurora B to kinetochores in RPE-1 cells (von Schubert et al., 2015). However, it is important to note that PLK1 kinase inhibition only partially reduces Aurora B activity and kinetochore localisation, consistent with the existence of multiple and redundant mechanisms promoting Aurora B tethering involving Haspin phosphorylation of Histone H3 T3 and BUB1 phosphorylation of T120H2A, that recruits Borealin through Shugoshin (Chu et al., 2011; Dai et al., 2005; Kawashima et al., 2010; Tsukahara et al., 2010). The presence of the multiple redundant pathways which may vary in relevance in different cell types may account for the discrepancies in the above-mentioned reports.

We show that Haspin activity is reduced when PLK1 is inhibited which is consistent with published work (Zhou et al., 2014). This suggests that the effect of PLK1 inhibition on Aurora B and its activity under our experimental conditions may be at least partially mediated through Haspin activity at the kinetochore. Similarly, PLK1 activity is continuously required for its own tethering at kinetochores which is in line with previous work demonstrating that PLK1 phosphorylates PBIP1 at T68 to create a phospho-binding domain for the Polo Box Domain of PLK1 itself (Kang et al., 2006).

We find that outer kinetochore proteins, ZWINT-1 and HEC1 are mis-localised when PLK1 is inhibited. This data indicates that inhibition of PLK1 or Aurora B causes incomplete kinetochore recruitment of HEC1 and ZWINT-1, which may be required for accurate recruitment of PLK1 and maintenance of Aurora B at the kinetochore. The large PLK1 foci are sometimes in proximity to CREST but are often found outside of chromosomes. This may be because the outer kinetochore proteins are not properly recruited under these conditions and PLK1 may require correct outer kinetochore protein localisation for it to be accurately maintained at the kinetochore and possibly to prevent PLK1 protein aggregation.

More surprisingly, we found that Hesperadin, an inhibitor that has been described as specific for Aurora B (Hauf et al., 2003), reduces PLK1 tethering at most kinetochores. It has been reported that, at least in *Drosophila,* Aurora B contributes to PLK1 activity by phosphorylation of the PLK1 T-loop (Carmena et al., 2012a). However, recent work has revealed that the main kinase phosphorylating the PLK1 T-loop at T210 in unperturbed mitosis in human cells is Bora-Aurora A (Bruinsma et al., 2014). We observed that in nocodazole treated cells, overall phosphorylation of PLK1 at T210 is indeed reduced by Hesperadin treatment suggesting that Aurora B may be partially responsible for maintaining PLK1 activation under these experimental conditions. This can occur by direct phosphorylation or indirectly by counteracting a PLK1 targeting protein phosphatase. Alternatively, the reduction in pT210-PLK1 may simply be related to PLK1 displacement from kinetochores and therefore the impossibility of being phosphorylated by a kinetochore resident kinase.

The similarities in the phenotypes observed upon PLK1 inhibition and Hesperadin-mediated inhibition of Aurora B, suggest that these kinases may be part of a positive feedback loop acting at kinetochores during SAC maintenance. This idea is reinforced by the observation that PLK1 inhibition, together with reduced Aurora B activity, has a strong effect in promoting SAC silencing consistent with recently published data under different experimental settings (Li et al., 2015). Intriguingly, some small molecule inhibitors of PLK1, including BI 6727 that was used in this study, can cause a mitotic arrest at their effective concentrations but at much higher doses were shown to mis-localise Aurora B from the kinetochore and result in SAC override (Raab et al., 2015). This is likely to be caused by off target effects that are revealed only when the compound is used at very high concentrations or, it is possible that PLK1 may have an important role in maintaining SAC signalling and that, under these checkpoint conditions, its ATP pocket may assume a different conformation, therefore the higher requirement of inhibitors to fully block PLK1 checkpoint function.

Finally, we were unable to confirm the recent finding demonstrating that MPS1 and PLK1 inhibition cooperates to cause checkpoint override (von Schubert et al., 2015). Intriguingly, we observed a trend in promoting checkpoint override but the data did not reach statistical significance. These discrepancies may be explained by either differences in PLK1 inhibitors used in the two studies (GW843682X and BI 6727 versus TAL) and/or the relevance of the two pathways in the different cell types (U2OS versus RPE1).

PLK1 inhibitors are being developed as anticancer agents and it is important to understand how these can be best used either as single agents or in effective combination therapies. We believe that this work contributes to our understanding of how PLK1 inhibition can affect the fate of cells arrested in mitosis and may provide a rational for testing mitotic kinase inhibitors in multidrug combination approaches.

## MATERIALS AND METHODS

### Cell culture

Osteosarcoma (U2OS) cells from Noel Lowndes Laboratory were first authenticated by STR analysis and subsequently certified by transposon profiling. Cells were cultured at 37°C, 5% CO_2_ in Dulbecco's modified Eagles medium supplemented with 1% penicillin-streptomycin and heat inactivated 10% foetal bovine serum (Sigma Aldrich, Wicklow, Ireland).

### Drugs and chemicals

Nocodazole (M1404; Sigma Aldrich) was used at 3.3 μM, MG132 at 10 μm (C2211, Sigma Aldrich), GW843682X at 1 μm (2977, Tocris, Bristol, UK), BI 6727 at 100 nM (S2235, Stratech Scientific, Suffolk, UK), Hesperadin at 100 nM or 500 nM (2096, Axon Medchem, Groningen, The Netherlands), Reversine at 100 nM or 500 nM (10004412, Cayman Chemical, Michigan, USA), 5-Iodotubercidin at 10 μm (HY-15424, Axon Medchem). DMSO was used as a control in the experiments.

### Antibodies

Primary antibodies used: mouse anti-AIM-1 (1:100; 611082, BD Biosciences, Dublin, Ireland), rabbit anti-pSer7CENP-A (1:200; 04-792, Millipore, Cork, Ireland), anti-centromere (1:100; 15-234, Antibodies Incorporated, CA, USA), mouse anti-PLK1 (1:200 for IF; ab17057, Abcam, Cambridge, UK), mouse anti-PLK1 (1:500 for WB; sc-56948, Santa Cruz, Heidelberg, Germany), rabbit anti-Histone H3 pSer10 (1:50; 06-570, Millipore), rabbit anti-GAPDH (1:1000; 14C10 Cell Signaling, MA, USA), mouse anti-HEC1 (1:500 for IF, 1:1000 for WB; clone 9G2.23, Genetex, Eching, Germany), rabbit anti-ZWINT-1 (1:100 for IF, 1:1000 for WB; IHC-00095, Bethyl, TX, USA), mouse anti-pT210-PLK1 (1:300 for IF, 1:1000 for WB; 39068, Abcam), pT3H3 (1:400 for IF 1:1000 for WB; 9714S, Cell Signaling), pMPM2 (1:1000; 05-368, Millipore), anti-mouse Cyclin B (1:1000; SC-245, Santa Cruz). Secondary antibodies used were Alexa Fluor 488 goat anti-mouse (1:300; A11001, Life Technologies, Dublin, Ireland), Alexa Fluor 546 goat anti-rabbit (1:300; A11010, Life Technologies), Alexa Fluor 647 goat anti-human (1:300; A21445, Life Technologies), goat anti-rabbit Alexa Fluor 488 (1:50 for FACS; A11008, Life Technologies), IRDye^®^ antibodies (1:10,000; LI-COR Biosciences, NE, USA).

### Flow cytometry

Cells were harvested and fixed with 70% ethanol, then permeabilised with 0.25% Triton-X 100 in PBS for 15 min on ice. Cells were incubated with primary antibodies for 1 h followed by incubation with secondary antibodies for 1 h. Cells were resuspended in PBS. Flow cytometry was carried out on a BD FACS Canto A and data was analysed using FlowJo software version 10.

### Immunofluorescence microscopy

Cells were fixed with 4% paraformaldehyde (PFA) for 10 min or for anti-PLK1 (Abcam), cells were fixed with 4% PFA in PHEM (30 mM HEPES pH 6.9, 65 mM PIPES, 10 mM EGTA, 2 mM MgCl_2_, 100 mM NaCl) plus 0.5% Triton X-100, and permeabilised with 0.1% Triton X-100. After blocking, cells were incubated with primary antibodies, followed by secondary antibodies. Nuclei were stained with DAPI and coverslips were mounted using SlowFade Gold Antifade Reagent (S36936, Life Technologies). Imaging was routinely performed on a Delta Vision microscope (Applied Precision, WA, USA) using a 100× objective lens, NA 1.40, and *z*-stacks taken every 0.3 μm across the cell. Images were deconvolved and projected using SoftWoRx (Applied Precision). Images in any particular figure were acquired using the same settings and were imported into Adobe Photoshop CS and pixel resolution and intensity levels were adjusted. Figures were assembled using Adobe Illustrator.

### High-throughput imaging and analysis

For quantification purposes, slides were imaged using a High Content Operetta system (PerkinElmer, London, UK) and a 60× WD objective lens. Between 42 and 72 fields of view were imaged per slide and 7 focal planes recorded for each field, with a distance of 1.2 µm between each plane, to ensure that the planes covered the height of the nucleus. Fluorophore excitation times were kept constant for all slides: 200 ms for DAPI (emission 410-480 nm), 400 ms for Alexa Fluor 488 (emission 500-550 nm), 600 ms for Alexa Fluor 546 (emission 560-630 nm) and 400 ms for DRAQ5 (emission 650-760 nm).

Images were analysed using Harmony 3.1.1 (PerkinElmer). Images from each field were processed as a stack, where 5 focal planes per field were stacked using the maximum projection tool.

Nuclei were detected using a Find Nuclei building block and the method C, with common threshold of 0.20, area >120 µm^2^, split factor of 10.0, individual threshold of 0.20 and contrast >0.0. To ensure only whole nuclei were analysed, border objects were excluded. Next, we used a combination of Morphology Properties, DAPI Intensity Properties and Number of DAPI spots to select mitotic cells only. The Morphology Properties (using the standard method) and the DAPI Intensity Properties were calculated for the remaining entire nuclei. A Find Spot building block was used to find spots in the DAPI channel, over the nucleus region of the Entire Cells population, using the method A, with Relative Spot Intensity >0.030 and a Splitting Coefficient of 1.0. To establish the Final Population of mitotic cells, a Select Population building block was used to include cells with the following characteristics: number of DAPI spots ≥15, nucleus area >120 µm^2^ and <650 µm^2^, and a Nucleus Ratio of Width to Length >0.4.

In our experiments, CREST, which localises to the centromere, was used as a localisation marker for pSer7CENP-A and Aurora B. We first used a Find Spots building block to identify CREST Spots in the DRAQ5 channel, over the nucleus area of the Final Population, using the Method A, with a Relative Spot Intensity >0.0 and Splitting Coefficient of 1.0. We next looked for Aurora B spots and pSer7CENP-A spots, within the area of the CREST spots. For this, the size of the CREST spots was increased by 2 pixels using a Select Region building block. Two Find Spots building blocks were used to look for Aurora B spots in the Alexa Fluor 488 channel and pSer7CENP-A spots in the Alexa Fluor 546 channel, both within the resized CREST spots, using the Method A, with Relative Spot Intensity >0.0 and a Splitting Coefficient of 1.0.

This allowed us to match each Aurora B and pSer7CENP-A spot to its original CREST spot. The outputs were Single Cell results for all properties calculated in the Analysis Sequence.

Finally, to address the effect of Aurora B and PLK1 inhibition on the localisation of Aurora B and pSer7CENP-A at the centromere, we looked at the fluorescence intensity of each Aurora B and pSer7CENP-A spot, after standardising it to the fluorescence intensity of its original CREST spot: following Harmony analysis, single cell results were used to match Aurora B and pSer7CENP-A spots to corresponding CREST spots, and to calculate the ratios of Corrected Spot Intensity pSer7CENP-A/CREST and Aurora B/CREST for each spot. The average ratio per treatment was then calculated. At least 680 mitotic cells per treatment were included in the counts, and the number of CREST spots was at least 13,900 per treatment. Data shown are the mean and s.e.m. of three independent experiments. For the PLK1 foci quantification in mitotic cells, slides were imaged and analysed using the above protocol, and PLK1 spots were identified in the 488 channel using the method C.

### Differential interference contrast (DIC) microscopy

Cells were captured using an Olympus CKX41 microscope with a CAchN 10× objective, 0.25 PhP and 0.30 NA with CellSens Entry software.

### Statistical analysis

Statistical analysis was performed using Microsoft Excel and GraphPad Prism software. Three experimental repeats were performed for each dataset. Data are presented using either s.e.m. or s.d. as indicated in the figure legends. Student's *t*-test was used to determine statistical significance between two groups.

### Protein manipulation

For western blotting analysis, cells were lysed in NP-40 buffer containing 50 mM Tris pH 7.5, 300 mM NaCl, 10 mM MgCl_2_, 0.1% NP-40, protease and phosphatase inhibitors (Sigma Aldrich) or CSK buffer containing 10 mM PIPES pH 6.8, 300 mM NaCl, 300 mM sucrose, 1.5 mM MgCl_2_, 10 mM NaF, 1 mM DTT, 0.5% Triton X-100, protease and phosphatase inhibitors. Protein concentrations were determined by Bradford assay. Cell extracts were resolved by SDS-PAGE, blotted onto nitrocellulose membranes, were probed with primary antibodies as indicated and IRDye^®^ secondary antibodies and were used for detection on an Odyssey infrared imaging system (LI-COR).
